# Thermal scanning probe lithography—a review

**DOI:** 10.1038/s41378-019-0124-8

**Published:** 2020-04-06

**Authors:** Samuel Tobias Howell, Anya Grushina, Felix Holzner, Juergen Brugger

**Affiliations:** 1 Microsystems Laboratory, École Polytechnique Fédérale de Lausanne (EPFL), 1015 Lausanne, Switzerland; 2 Heidelberg Instruments Nano - SwissLitho AG, Technoparkstrasse 1, 8005 Zürich, Switzerland

**Keywords:** Nanoscale devices, Nanoscale materials, Chemistry, Optical materials and structures, Nanoscale materials

## Abstract

Fundamental aspects and state-of-the-art results of thermal scanning probe lithography (t-SPL) are reviewed here. t-SPL is an emerging direct-write nanolithography method with many unique properties which enable original or improved nano-patterning in application fields ranging from quantum technologies to material science. In particular, ultrafast and highly localized thermal processing of surfaces can be achieved through the sharp heated tip in t-SPL to generate high-resolution patterns. We investigate t-SPL as a means of generating three types of material interaction: removal, conversion, and addition. Each of these categories is illustrated with process parameters and application examples, as well as their respective opportunities and challenges. Our intention is to provide a knowledge base of t-SPL capabilities and current limitations and to guide nanoengineers to the best-fitting approach of t-SPL for their challenges in nanofabrication or material science. Many potential applications of nanoscale modifications with thermal probes still wait to be explored, in particular when one can utilize the inherently ultrahigh heating and cooling rates.

## Review: Thermal scanning probe lithography

Thermal scanning probe lithography is reviewed in the context of material removal, conversion and deposition. Scanning probe lithography has long been a promising technique for direct-write nanoscale patterning on surfaces. However, while the technique is widely used in research labs, the slow write speed has limited its use in industrial settings. Instead, thermal scanning probe lithography has emerged, in which a heated tip is used to induce localized changes in the material, enabling write speeds limited by the speed of movement of the tip itself. A team from École Polytechnique Fédérale de Lausanne led by Juergen Brugger now reviews the current state of play for thermal scanning probe lithography, focusing on whether material is removed, changed or deposited by the heated tip, and the types of materials that have been studied.

## Graphical abstract

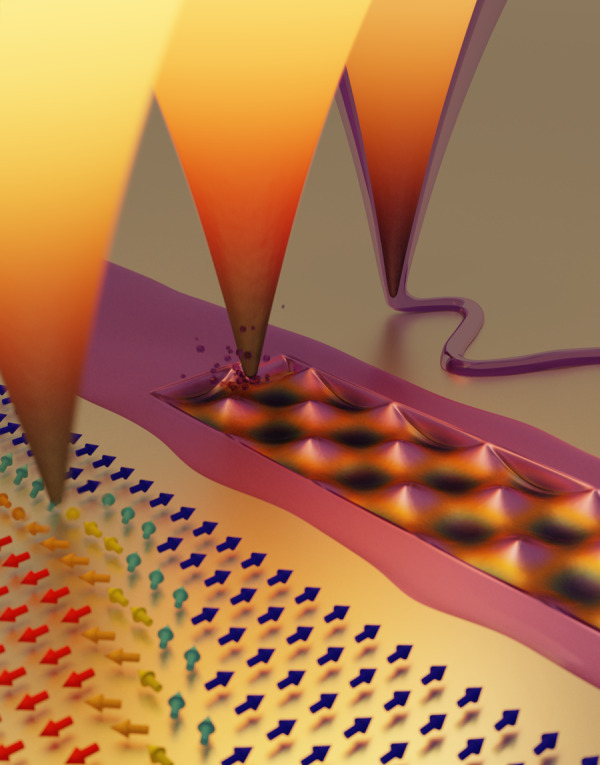

## Introduction

Throughout human history, heat has always held a key role in manufacturing processes. Starting with clay firing more than 26,000 years ago^1^, many heat-related manufacturing processes have since then been developed, such as casting, sintering, and molding. Heat can be used to shape, modify, and create a broad range of materials, including ceramics, metals, semiconductors, and polymers. Traditional thermal treatments involved the use of furnaces or hot plates, but with increasing manufacturing complexity, heat is more often locally applied, e.g., in soldering or laser-induced processing. Additive manufacturing techniques, such as 3D printing, rely on localized heat to create structures of almost any shape, e.g., to soften thermoplastics for extrusion or to sinter granular materials. On the sub-micron scale, data within DVDs and Blu-Ray disks can be saved and modified through heat-induced phase changes by a focused laser.

Many microsystems and nanosystems require precisely fabricated nanoscale patterns that exhibit an intrinsic functionality, such as certain electronic, photonic, chemical, and mechanical properties. To fabricate these nanoscale patterns, electron-beam lithography (EBL) is the most established direct-write, mask-less lithography technique. EBL tools are the workhorses for nanofabrication in R&D facilities and for manufacturing high-end industrial photomasks. However, EBL is relatively expensive because it requires complex electron-compatible optics to focus the electron beam into a spot of a few nanometers. Another issue is electron scattering, a type of proximity effect, on the sample surface, which leads to an additional undesired resist exposure that has to be corrected by computing-intensive algorithms.

Scanning probe lithography (SPL) is another direct-write nanolithography technique, where patterns are created by scanning a nanometer-sharp tip over the sample to locally induce modifications. Tip-sample interactions are manifold and can include mechanical, electrical, diffusive, and thermal effects. SPL methods are intensively studied and have already been reviewed in the literature^2–4^. Despite the fact that some SPL techniques with nanometer resolution were already demonstrated 30 years ago^5^, today, SPL techniques are primarily used in academic research. This can be attributed to the slow writing speed of some SPL techniques, on the order of 0.1–50 μm/s in the case of oxidation SPL^6^.

One type of SPL technique, thermal scanning probe lithography (t-SPL), has recently reestablished itself as fast and reliable; in this technique thermal energy from a heated tip is used to induce local material modifications. Heat is a universal stimulus for material conversion and can induce crystallization, evaporation, melting, etc. in a variety of materials. At the nanometer scale, where only small volumes are heated, characteristic time scales are on the order of nanosceconds. Hence, heating for few microseconds is sufficient to modify material under the tip and the mechanical scan movement of the cantilever becomes the main limitation in regard to the writing speed. Writing speeds of up to 20 mm/s have been achieved with a single tip at a pixel rate of 500 kHz^7^.

**Fig. 1:**
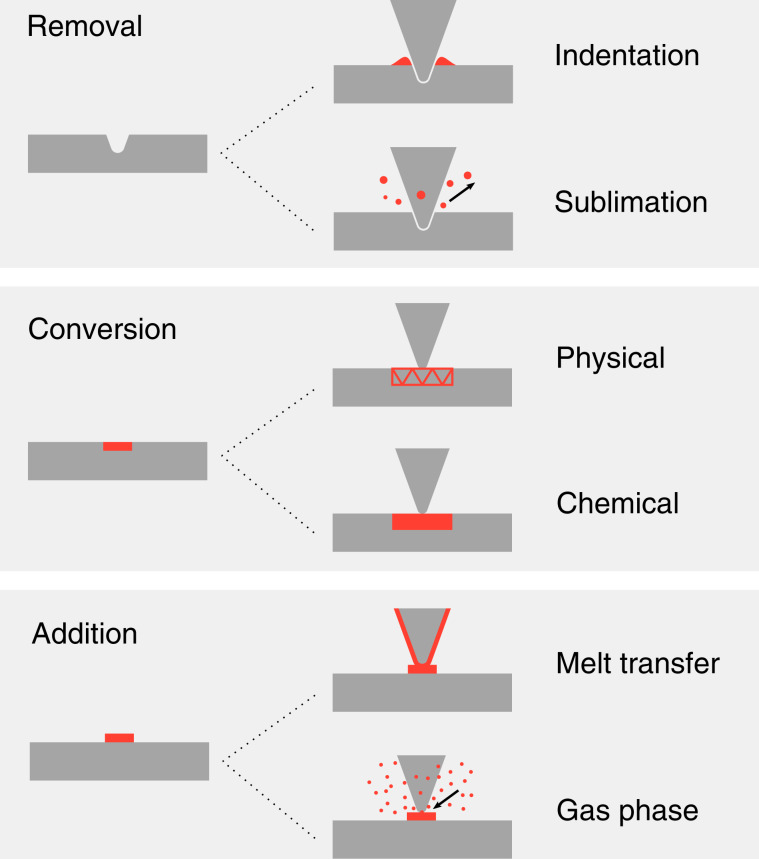
Overview of concepts of local modifications induced by a thermal scanning probe on a sample. Removal of material by thermomechanical indentation or sublimation of a sample material; Conversion of a sample by local modification of its physical properties such as the crystallinity or magnetic dipole orientation, or chemical conversion; Addition of a functional material by melt transfer from a heated tip to the substrate or from a gas phase such as chemical vapor deposition of a precursor material

To date, t-SPL has reached a high level of technical maturity and dedicated tools exist to perform reliable thermal nano-lithography, which is reflected in the increasing number of scientific publications related to the topic. This article provides an overview of the most recent t-SPL techniques and provides an exhaustive list of materials that have been modified or deposited through t-SPL. We also discuss relevant fabrication processes developed or adapted for t-SPL that were not covered in previous articles^2,3,6^. t-SPL techniques have been categorized into the removal, conversion, and addition of material, as illustrated in Fig. 1. In Table 1, the most important characteristics and figures-of-merit for each technique are summarized, while in Table 2, a comprehensive overview of materials that have been investigated in t-SPL is provided. Only materials that have been directly modified with a thermal probe are listed but many more can be modified by subsequent processing steps (etching, plating, molding, lift-off, etc.). Each category of t-SPL is discussed separately within the following text to highlight their own unique advantages and specific challenges. The illustrations and tables, combined with references to primary literature, aims to guide the reader to find out how a particular nanofabrication challenge can be best addressed by t-SPL.

**Table 1:** Comparison of the three concepts for thermal scanning probe lithography: removal, conversion and addition of material

	Removal	Conversion	Addition
Resolution			
Demonstrated	8 nm half-pitch^29,128^	10 nm feature size^81^	35 nm^110^ 10 nm^117^ (with particles)
Typical	20–50 nm	50–100 nm	100–500 nm
Limitation	Tip shape, temperature	Tip shape, temperature	Large effective tip radius due to loaded material, substrate wettability, temperature, contact duration
Speed			
Demonstrated	20 mm/s^7^	1.4 mm/s^78^	40 µm/s^110^
Typical	0.1–1 mm/s	1–200 µm/s	0.1–5 µm/s
Limitation	Actuation frequency, and trade-off between scan speed and position accuracy	Reaction kinetics, heat diffusion	Mass flow, diffusion
Features and challenges	Closed-loop lithography permits 3D (grayscale) lithography with 1 nm vertical resolution Sub-10 nm marker-less overlay and field stitching Long tip lifetime possible (few days patterning), despite potential challenges with tip contamination	New material systems can be experimentally tested very quickly if suitable for conversion Strong temperature/force dependence can limit the reproducibility Patterning on hard surfaces can cause severe tip wear	Resolution depends on various parameters that need to be controlled well Risk of contaminating sample with ink during imaging Ink supply limited by tip capacity (might require reload of tip)
Applications	Prototyping of all kind of nanodevices and 3D masters Wide selection of etch transfer, lift-off and other processes for almost any material Compatible with mix-and-match lithography with integrated laser for increased throughput	Large potential for biological applications that require specific chemical binding Unique capability to produce nanoscale chemical gradients Nanodevices that require local change of physical properties like magnetization, ferroelectricity or conductivity	Deposition of various polymers, composites and metals Direct deposition of polymeric masks for further standard nanodevice processing Suitable for devices with topographies where negative resist patterns are required
Maturity	Technologically advanced (dedicated machines, cantilevers, softwares, resists, and processes) Alternative and extension to standard electron beam lithography in particular for materials/devices sensitive to damage from charged particle beams	Wide range of materials that can be thermally modified Few established processes beyond the individual research groups who developed it Good calibration and understanding might be required	Exploratory status Few people with experience Tip loading, good calibration and understanding of the deposition process are required

Note that the values for resolution and speed need to be interpreted with great care. Best demonstrated values often mean that experiments are pushed to the extreme and at the expense of other criteria, while best values also don’t necessarily mean that these values are already the physical limits. We quote the best demonstrated and typical values found in literature and used for real applications and add an interpretation based on our own experience and assumptions

**Table 2:** Overview of materials patterned by t-SPL with a brief description and the relevant references

Material	Comment	Ref.
Removal		
CSAR 62	A commercial EBL resist which can be directly removed by a heated tip. High contamination of the tip	Personal communication to the author
Diels-Alder polymer	Fast (~10 µs) and reversible cross-linking upon heating	^129^
Fluorocarbon	Thermomechanical indentation of the thin film (0.5 µm/s)	^130^
Diphenylalanine nanotubes	Thermomechanical machining of nanotubes	Personal communication to the author
Molecular glasses (various)	Desorbs from substrate upon heating. High resolution possible. Can be deposited by evaporation.	^48–50^
Polyaryletherketone (PAEK)	Thermomechanical indentation in highly cross-linked polymer	^15^
Polycarbonate (PC)	Cross-linked PC used for thermal degradation with heated tip (0.5–3 µm/s)	^9,46^
Pentaerythritol tratranitrate (PETN)	Thermal degradation of an energetic material leading to much wider structures with higher temperatures	^47^
Polymethylmethacrylate (PMMA)	Indentation, thermal degradation (10 µm/s)	^8,11–13,51,131,132^
PMMA/MA	Serves usually as underlayer for bilayer lift-off but also possible to remove by heated tip	Personal communication to the author
Polyphthalaldehyde (PPA)	Most used t-SPL resist, clean decomposition at glass point, sub-10- nm resolution, accurate grayscale and high speed demonstrated	^7,29–31,33,36–39,43,52–77^
Polystyrene (PS)	Indentation	^16–18,133^
Polysulfone	Ripple formation study	^17,134^
Polythioaminal	Resist for sublimation via thermal decomposition	^126^
SU-8	Thermomechanical indentation	^135^
Conversion		
3-mercaptopropyltriethosysilane (MPTES)	Deprotection of THP group (3 µm/s)	^83^
Aminopropyltriethoxysilane (APTES)	Surface activation	^83^
AZ 5214E	Thermally activated crosslinking (10–20 nm/s)	^87,95^
BiFeO_3_	Thermally induced crystallization of ferroelectric nanodots from sol–gel prepared film	^100^
Co/Pt	Thermally-assisted magnetic recording	^136^
CoFe_2_O_4_	Thermally induced crystallization of ferromagnetic nanodots from sol-gel prepared film	^101^
GeSbTe	Phase-change material crystallized by 100 ns heat pulses and rapid cooling	^21^
GeTe	Crystallization of material upon heating (<1 µm/s)	^98^
Graphene	Oxidation of graphene with a heated probe in oxygen-containing atmosphere	^87^
Graphene oxide	Thermal reduction of graphene oxide for nanoelectronics	^42,45,86^
Graphene fluoride	Thermal reduction of graphene fluoride (10–50 nm/s)	^137^
InSnSb	Tip-induced crystallization of phase change material	^9^
p(THP-MA)_80_-p(PMC-MA)_20_	Thermal deprotection of functional amine groups, chemical gradients possible (<1.4 mm/s)	^32,33,78–82,138,139^
Poly(tert-butyl acrylate)	Thermal deprotection of tert-butyl ester groups uncovers carboxylic acid moities (5–500 µm/s)	^84,85^
PbTiO_3_ / PbZrTiO_3_ (PZT)	Ferroelectric nanostructures from sol-gel precursor (contact duration 0.6 s)	^99^
Pentacene precursor	13,6-N-Sulfinylacetamidopentacene, for organic nanoelectronics	^88,92^
Polyarylenetriazolylene (PArT) precursors	“Click Chemistry” thermally triggered 1,3-dipolar cycloaddition to fabricate organic semiconductors (16 µm/s)	^94^
Polyethylene terephthalate (PET)	Crystallization of amorphous PET by thermal annealing with a heated probe	^140^
PMMA/BPA/Ag_3_N	Thermally triggered exothermal reaction, increases accessible temperature	^141^
Poly(p-phenyene vinylene) (PPV) precursor	Nanoscale conducting polymer structures from a precursor film (<80 µm/s)	^42,88–91^
PVDF-TrFE	Thermally induced crystallization of ferroelectric nanodots	^102^
Ru/IrMn/CoFeB	Anti- and ferromagnetic multilayers to fabricate nanoscale magnetic structures	^103–106^
TbFe	Thermally-assisted magnetic recording	^136^
Titanium	Thermal oxidation with a heated probe	^87^
Silk fibroin	Solubility contrast in water by fast melting of beta sheet crystallites	^97^
Supramolecular glass	Supramolecular polymers can reversibly form aggregates upon heating	^96^
Addition		
Cu, CuO	Tip-based chemical vapor deposition from copper acetylacetonate precursor	^124,125^
Indium	Direct fabrication of indium oxide nanowires	^116^
Mercaptohexadecanoic acid (MHA)	Study of temperature dependence of ink transport	^109^
Octadecylphosphonic acid (OPA)	Self-assembled monolayer (1 µm/s)	^111^
Poly(3-dodecylthiophene) (PDDT)	Deposition of self-assembled monolayers of a conducting polymer (10 µm/s)	^112–114,117^
Polyethylene (PE)	Direct deposition as etching mask and carrier of nanoparticles	^108,117^
Poly(N-isopropylacrylamide) (PNIPAAm)	Deposition of functional polymer	^115^
Poly(methyl methacrylate) (PMMA)	Use as a mask for XeF_2_ etching of MoS_2_ nanoribbons	^120^
Polystyrene (PS)	Use as a mask for dry etching	^110,118,121,122^
PVDF-TrFE	Direct deposition of a ferroelectric polymer with and without metalorganic additives	^117^

### A brief history of t-SPL

The first experiments with heated atomic force microscopy (AFM) tips were reported by IBM Research Laboratories with the intention of applying scanning tip-based technologies for data storage applications. This early exploratory work involved the actuation of a conventional AFM cantilever, which is heated by a laser, to create nanoscale indents into a rotating poly(methyl methacrylate) (PMMA) disk^8^. Further improvements included integrating a resistive heater in the AFM cantilevers above the AFM tip to avoid the external laser heating^9,10^ and developing the integrated resistive heaters into distance sensors to measure the vertical cantilever position with respect to the sample^11^. Major advances in the development of thermal probes were achieved during the so-called “Millipede project” at IBM, where parallel arrays of up to 4096 independently-controlled thermal cantilevers were developed to enable high-speed writing, reading, and erasing of data indents^12–14^. At the same time, the polymer recording media was optimized to improve formation and erasing of the data indents and to increase the tip lifetime by minimizing wear^11,15–18^.

Although in 2002, the IBM’s Millipede project was holding records for areal densities, it nevertheless did not become commercialized^19^. Despite this, the key technologies developed during the Millipede project, such as integrated heating, thermal topography sensing and electrostatic tip actuation, are now implemented in modern cantilevers for t-SPL.

**Fig. 2:**
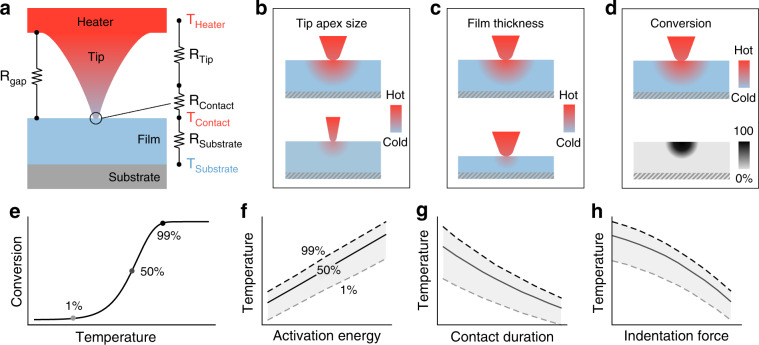
Heat transfer model and parameters affecting temperature and nanoscale thermal reaction kinetics under the tip. **a** Heat transfer model of a resistively heated cantilever: Heat is conducted from the source through the tip and the air gap into the sample material and the substrate. **b** Effect of tip apex size and opening angle on the tip-sample contact temperature and resolution. Typically, with larger tip opening angles and large apex radius, a higher contact temperature can be achieved at the cost of resolution. **c** Effect of film thickness on the temperature distribution for a typical situation where the thermal conductivity of the film (e.g., polymer) is lower than the substrate (e.g., Si). The tip-sample contact temperature decreases with decreasing film thickness. **d** Qualitative sketch of how the temperature distribution in the material relates to the corresponding converted volume of material in the film. For most reactions relevant to t-SPL, the thermally converted volume is smaller than the spreading of heat, which benefits the lateral resolution. **e** Plot of a first-order reaction where the fraction of converted material with respect to the total material is plotted as a function of the tip temperature. The points where 1%, 50% and 99% of the material is converted are indicated. The following plots show how the temperature is affected by activation energy, tip-sample contact duration and indentation force. **f** When the activation energy of a thermally driven process increases, a higher temperature is required to induce a material modification. **g** The longer the tip is in contact with the material, the lower the temperature required to induce a modification and the narrower the temperature range over which the conversion takes place (*x*-axis is logarithmic). **h** The temperature required for certain chemical reactions to complete can be lowered by increasing the pressure. In t-SPL, this can be achieved by increasing the indentation force

Most of today’s t-SPL tools control the tip temperature via a resistively heated element incorporated into the cantilever above the tip^20^ as illustrated in Fig. 2a. Owing to the small size of the heating element (few micrometers) and the cantilever (tens of micrometers), the cantilever’s thermal constant is on the order of a few microseconds, which produces ultrafast heating and cooling^7^. Alternatively, a laser can be used to heat the tip by directly illuminating either the backside of the cantilever or the tip apex so that a fraction of the laser energy is absorbed and converted into heat^8,21^. Tip-assisted laser ablation is another approach for nanopatterning materials but is not discussed in this work^22–24^. The different working principles of cantilevers for t-SPL are comprehensively reviewed elsewhere^20,25^.

The first commercialized t-SPL system became available in 2014, which has revitalized interest in thermal nanolithography and fostered research in many different materials’ systems and pattern transfer processes. As will be discussed in depth below, in some applications, t-SPL’s unique capabilities surpass other lithography methods and enable various new applications for nanotechnology.

### Strengths of t-SPL

t-SPL combines the following advantages:

- Heat as a stimulus: Heat is a universal stimulus and induces functional modifications in a wide range of materials.
- No charged particles: t-SPL induces material modifications via phonons instead of exposure to charged particles such as electrons or ions, thereby avoiding unwanted creation or scission of covalent bonds, lattice defects, vacancies or trapped charges. This aspect becomes increasingly important for devices made of low-dimensional materials such as graphene^26,27^ or MoS_2_^28^. Charged particle beams also lead to proximity effects that are difficult to correct for complex geometries at high resolution.
- Sub-10 nm resolution: A heated tip with an apex diameter below 10 nm can produce complex geometries with a lateral resolution below 10 nm^29^, which is more than one order of magnitude better than direct-write laser lithography.
- 3D patterning: Today’s t-SPL tools precisely control the actuation force and the tip-sample contact duration, thereby enabling 3D (grayscale) patterning^30^ with vertical resolution better than 1 nm^31^ and controlled patterning of chemical gradients^32,33^.
- Robust and compact setup: The components of a t-SPL tool are relatively simple and cost-effective compared to focused electron or ion beam systems. Because the tools are very compact, they can be placed almost anywhere, e.g., inside a glovebox for patterning in inert gas environments. At the same time, t-SPL is compatible with ambient environments.
- In situ AFM imaging and closed-loop lithography: In addition to patterning, the t-SPL tip can image the surface topography before, after and during patterning, similarly to an AFM tip (with the tip heater turned off). Hence, the quality of the written pattern can be imaged in situ, which saves time and cost during development of new processes. To maintain a high t-SPL patterning quality during writing (e.g., the correct patterning depth in grayscale lithography), software algorithms compare the actual and the target topography during writing and adapt patterning parameters such as the actuation force. In addition, closed-loop lithography helps to actively compensate for potential changes of the tip (wear or contamination), the sample (e.g., varying thermal properties) or the environment (e.g., thermal drifts)^34,35^.
- Overlay and stitching without markers: The in situ inspection capability enables precise overlay and marker-less stitching. Samples can be imaged before the patterning process to locate previously patterned structures, nanowires^36^ or 2D flakes. An overlay onto structures buried under the resist can be done without artificial markers with sub-5 nm precision^37,38^. Moreover, the natural sample surface roughness can be imagined and used as unique marker for stitching of multiple patterning fields at an offset error smaller than 10 nm^39^.

### Challenges in t-SPL

The following challenges currently limit the application range of t-SPL:

- Throughput: Depending on the induced thermal modification, the patterning speed in t-SPL is either limited by the reaction kinetics or by the mechanical movement of the tip (e.g., slow recrystallization vs. fast self-amplified depolymerization). Mechanics limit the practical scan speed of an individual tip to few mm/s^7^. This speed is comparable to that of EBL or ion beam lithography operating at their highest possible resolution, which is limited to less than 1 mm/s due to low beam currents and high required doses for high-resolution resists like HSQ^40^. Nowadays, high-resolution EBL mask writers use more than 250,000 electron beams in parallel in order to achieve the required throughput and resolution for high-end photo masks^41^. Similarly, using multiple tips in parallel ultimately could overcome the throughput limit for t-SPL^13,42^. Another strategy to increase throughput for applications that require microscale and nanoscale structures is to combine a high-resolution thermal scanning probe with a fast integrated laser writer in a single tool^43^.
- Tip degradation: Tip deterioration due to friction and contamination during patterning and imaging strongly depends on the tip and sample materials as well as the operation mode (sliding vs. indentation). The attainable resolution reduces with tip use, which affects the heat transfer from the tip to the sample. While tip wear is irreversible, some tip contaminations such as organic residues can be evaporated by excessive heating. Nevertheless, high-resolution patterning requires regular exchange of the cantilevers.
- Tip-sample contact temperature: The small tip-sample contact area makes it difficult to precisely determine the contact temperature. Calculations require knowledge of the thermal boundary resistance, which depends on the materials involved, the tip geometry and the roughness of the tip-sample interface. These parameters are not often experimentally available. Moreover, the tip-sample contact area and roughness can change during patterning. This restriction is less of an issue for applications that do not require high temperature or precise knowledge of the tip-sample contact temperature, because the optimal patterning parameters can be directly determined from the sample response using the in situ AFM capability of the t-SPL tools.

### What parameters influence the temperature and reaction under the tip?

In t-SPL, the temperature is the key parameter governing material modifications and determines the attainable resolution and patterning speed. Insufficient tip temperatures result in no or incomplete material modifications. Conversely, an excessively high temperature may reduce the resolution due to spreading of heat and may damage the sample material.

Figure 2a illustrates a common situation where the heated tip is in contact with a sample film that is coated onto a substrate. The sketch shows the thermal resistance model for the heat flow from the probe to the substrate^20,25^. This model is valid only for steady-state heat transfer, which holds true if the tip-sample contact duration is much longer than the characteristic time for diffusion of heat.

In the following list, we enumerate the most important factors in t-SPL that are related to temperature and provide a qualitative visualization in Fig. 2b–h. A more detailed discussion of their interplay is included in the supplementary information.

- Tip diameter and shape: The smaller the tip-apex diameter is, the better the achievable spatial resolution (Fig. 2b). However, the heat transfer through the tip is strongly reduced due to boundary scattering of phonons once the tip diameter is smaller than the mean free path of phonons (in silicon ~70–140 nm)^25,44^. Therefore, sharp tips with small opening angles achieve a lower tip-sample contact temperature than blunt tips with wide opening angles.
- Substrate thermal conductivity: The higher the thermal conductivity of the substrate or the sample material, the more efficiently the heat is transported away from the heat source. Consequentially, lower tip-sample contact temperatures occur in materials with a high thermal conductivity. A strategy to deal with this challenge is to warm up the entire sample with an external heat source (e.g., hot plate) to a set temperature.
- Sample film thickness: For thin films on a bulk substrate, the influence of the substrate thermal conductivity on the tip-sample contact temperature depends on the film thickness (Fig. 2c). As a rule of thumb, for a film thickness ten times larger than the tip apex diameter, the influence of the substrate thermal conductivity can be neglected^44^. For this reason, temperature-sensitive samples can be protected from the hot probe by a thick resist layer (typically >50 nm) or an intermediate layer.
- Indentation depth (for t-SPL processes that involve indentation): The deeper the tip penetrates into the sample, the larger the tip-sample contact area is. The extent of this effect strongly depends on the materials, the tip and the sample geometry: (i) for shallow indentation, the contact temperature increases with increasing indentation depth because more heat is transferred through the tip-sample contact area (thermal contact resistance is dominant), (ii) for deep indentation, the contact temperature decreases because tip cooling through the tip-sample interface becomes dominant (thermal spreading resistance is dominant). As an outcome, the heating efficiency first increases and then decreases as a function of indentation depth.
- Activation energy: The activation energy, defined as the potential barrier for the heated atoms or molecules to overcome, determines the temperature sensitivity of the reaction rate and how much material is converted at a specific temperature (Fig. 2d, e). The higher the activation energy of a reaction, the higher the temperature necessary to complete it (Fig. 2f). As a general rule, reactions with low activation energy, typically between 1 and 1.7 eV, are better suited for t-SPL (see supplementary information). As a positive side effect, the lateral extent of a thermally activated endothermic reaction is smaller than the heat spreading, which benefits the resolution of t-SPL^33^ as depicted in Fig. 2d.
- Tip-sample contact duration: The tip-sample contact duration directly affects the degree of conversion of a reaction (Fig. 2g). The longer the tip remains in contact with the sample, the more thermal energy is transferred. It is important to note that the tip-sample contact duration necessary to induce a material modification can be as short as a few microseconds and heating rates can reach >10^8^ K/s.
- Contact force/pressure: The tip-sample contact force affects the temperature and, potentially, the reaction kinetics of the t-SPL process in two ways: (i) a higher force on the cantilever increases the effective tip-sample contact area for more efficient heat transfer as discussed above and (ii) the contact force exerts a local pressure in the material under the tip, which can affect the reaction kinetics itself by reducing the reaction activation energy (Fig. 2h)^45^.

## Material removal

**Fig. 3:**
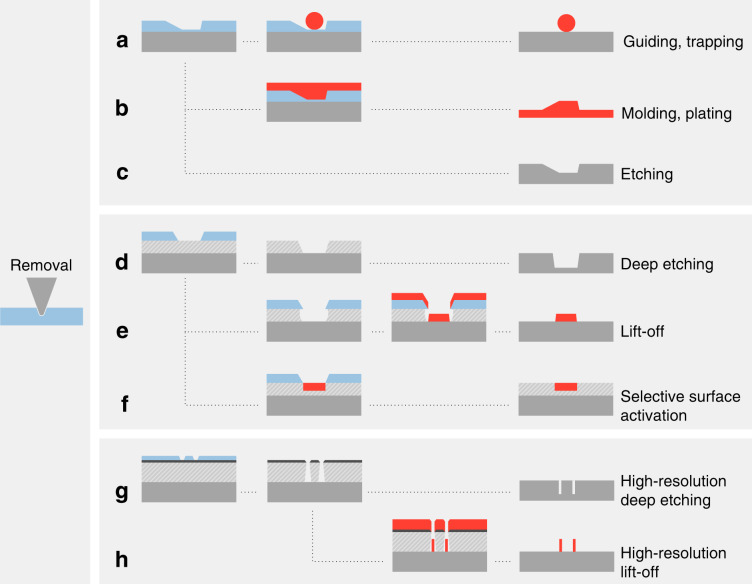
Overview of relevant nanofabrication processes after local removal of a temperature-sensitive resist. **a** 2D or 3D structures can directly serve as a biocompatible scaffold^59^ or be used to guide and trap nanoparticles^56,57,58^. After assembly, the PPA resist can be removed by heating^55^. **b** Molding of various soft transparent polymers directly from the patterned resist, as well as galvanic plating of metals, is possible^31,60^. **c** Direct use of the patterned resist as an etch mask allows etch transfer (wet or dry etch) of 2D and 3D features into various materials^54,61,63–65^. **d** A second layer under the temperature-sensitive resist can be used to amplify the final etch depth as well as for 3D features^67,66^. **e** A second organic layer under the temperature-sensitive resist that is selectively removed in wet chemistry can be used to create an undercut for lift-off^68,69^. **f** A functional layer under the temperature-sensitive resist can be locally accessed when the resist is removed and simultaneously activated by the thermal probe^70–72^ or in a subsequent step by oxygen plasma^73^. **g** A three-layer stack composed of a temperature-sensitive resist, a thin inorganic hard mask and an organic transfer layer is suitable for high aspect ratio and high-resolution etching^29,43,69,74–76^. **h** The three-layer stack can also be used for a high-resolution lift-off processes^76,77^

**Fig. 4:**
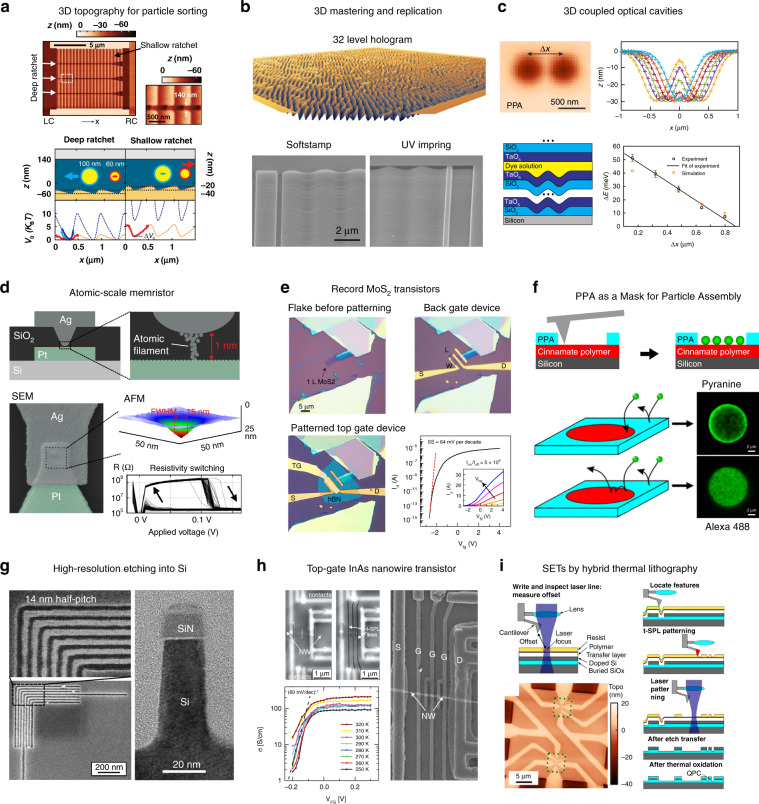
Examples of applications achieved by material removal using t-SPL. **a** 3D nanofluidic rocking Brownian motor sorting device for nanoparticles patterned into PPA resist. Reprinted with permission from ref. ^56^, AAAS. **b** Top: 32-level hologram in PPA, Bottom: a 300 nm deep sine wave used as master for UV nanoimprint. Reprinted with permission from ref. ^31^. **c** Gaussian-shaped optical microcavities patterned into PPA and etched into a Bragg mirror form photonic molecules. Adapted under CC BY 4.0 License from ref. ^54^, Copyright 2017 Springer Nature. **d** Atomic scale memristor devices fabricated by etching a cone-shaped t-SPL indent from PPA into SiO_2_. Adapted under CC BY 4.0 License from ref. ^65^, Copyright 2019 Springer Nature. **e** Single-layer MoS_2_ top gate transistor with record low contact resistance and on-off ratio. Reprinted with permission from ref. ^68^, Copyright 2019 Springer Nature. **f** Selective removal of PPA for accessing a functional material to study nanoparticle assembly processes. Reproduced with permission from ref. ^72^, Copyright 2018 ACS. **g** Left: 14 nm half-pitch pattern etched into Si. Reproduced with permission from ref. ^29^, Copyright 2017 ACS. Right: TEM image of a Si fin etched from patterned and Al_2_O_3_-infiltrated PPA resist. Adapted with permission from ref. ^75^, Copyright 2018 ACS. **h** InAs nanowire transistor overlaid with t-SPL-patterned metal top gate electrodes. Reprinted with permission from refs. ^76,77^, Copyright 2019 IEEE. **i** Integration of t-SPL with a laser writer for fabrication of Si-based room temperature single-electron transistors. Reproduced with permission from ref. ^43^, Copyright 2018 IOP Publishing

The most common use of t-SPL is localized material removal with the heated tip to modify the surface topography. We differentiate between mass-preserving thermomechanical indentation, where material is pushed to the side of each indent and permanent removal of material from the surface where material is sublimated, as depicted in Fig. 1. Even though indentation alone has little relevance for today’s t-SPL applications, materials used for thermomechanical indentation are included in Table 2. This section is dedicated to topographic patterning by permanent removal of polymer-based resists along with some concrete applications. After a discussion about the requirements on a good resist for permanent removal, we show examples of how topographic t-SPL patterns in a resist can subsequently be transferred into different materials by means of replication or transfer processes such as molding, etching and lift-off. Figure 3 illustrates pattern transfer processes from substrates covered by (a–c) a single resist layer, (d–f) a two-layer stack composed of a resist and an additional organic transfer layer, and (g, h) a three-layer stack composed of a resist, an inorganic hard mask and an organic transfer layer. Several relevant examples and applications of these fabrication processes are shown in Fig. 4.

### Requirements for t-SPL resists for direct permanent removal

Direct and permanent removal of material with a heated tip requires a transformation of the solid material into a gas. In t-SPL, formation of the liquid phase should be avoided because surface tension and material flow can deteriorate the pattern shape^46^.

t-SPL favors resist materials with a low activation energy for thermal degradation since the maximum thermal energy provided by the probe to sublimate a material is governed by the heat transfer. Therefore, the most suitable materials are organic resists. Good patterning performance can be obtained under the following conditions:

- Speed: Short contact duration (<100 µs) with the heated tip is sufficient to remove the resist.
- Resolution: Little resist reflow, no additional heat spreading from an exothermic reaction^47^, and mechanical stability of the written nanofeatures are advantageous.
- Coating: Smooth and thin films are readily deposited on many substrate surfaces.
- Contamination: No re-deposition of the removed resist or decomposed fragments onto the sample surface and no permanent contamination of the tip occur.

Molecular glasses are a class of materials that have been proven suitable for high-resolution t-SPL patterning^48–50^. They consist of small molecules that are weakly linked by hydrogen bonds and form a solid at room temperature. Short heat pulses by a heated probe are sufficient to locally sublimate the material. Molecular glasses can form clean films on many substrates through physical vapor deposition but have not been extensively used for t-SPL beyond proof-of-concept studies^48^. This is mainly because molecular glass resists tend to crystallize or take up water from the air. Furthermore, despite the specifically designed high glass transition temperature of molecular glass resists, the small molecules can feature a high surface mobility which affects the stability of the nanopatterns over time.

The example of molecular glass resists shows that a few additional requirements need to be considered for a t-SPL resist to be suited for general nanofabrication:

- Stability: The films and patterned nanostructures should be stable over a reasonable time period under ambient storage conditions.
- Compatibility with standard pattern transfer processes: Nanoscale patterns often need to be transferred into another material and hence the organic resist should be compatible with standard transfer processes such as etching, lift-off, molding and plating.
- Availability: Commercial availability with reliable quality and ideally with a shelf lifetime exceeding a few months are crucial.

Many polymer-based resists fulfill these additional requirements. However, only a few resists are well-suited for the permanent removal process by t-SPL, which requires that the chains of a resist are decomposed into volatile molecules at reasonably low temperatures and short timescales. Note that thermal degradation under short heat exposure, i.e. high heating rates, requires a temperature many times higher than the degradation temperature measured at slow heating rates (see Fig. 2g). Many conventional polymers such as polycarbonate (PC) or poly(methyl methacrylate) (PMMA) therefore require excessively high tip temperatures or long exposure times and begin to flow when heated above the glass transition, which limits their application range^46,51^. This does not exclude these polymers from being useful for t-SPL, but it may limit the resolution and the geometries that can be reliably patterned.

Currently, polyphthalaldehyde (PPA) is the most suitable and widely used temperature-sensitive resist for nanofabrication with t-SPL (see Table 2). The molecular structure of PPA is specifically tailored such that upon reaching the glass transition temperature (~150 °C) the polymer chains break and immediately unzip into small volatile molecules, so-called self-amplified depolymerization^52^. Consequently, PPA does not melt and flow, but rather directly sublimates^53^. In addition, the reaction is endothermic, which keeps the depolymerization localized. Due to this unique decomposition reaction, PPA has demonstrated sub-10 nm resolution^29^ and fast patterning (1 μs heating pulses)^7^. Due to the sublimation of the resist upon exposure with a heated probe, PPA can be removed without any exerted mechanical force and the resulting patterning depth is proportional to the cantilever deflection. Owing to this property, closed-loop lithography algorithms that rely on in situ measurement of the patterned structure topography enable creating grayscale geometries with deviations smaller than 0.7 nm (1*σ*) from the target depths^54^. The high precision and reliability of t-SPL using PPA resist enabled several novel applications discussed hereafter.

### Sublimation

#### Tailored topographies for guiding and assembly of micro-objects and nano-objects

Patterning continuous 3D (grayscale) topographies in PPA with nanometer vertical precision without residues or material flow is one of the unique features of t-SPL^52,55^. Proposed applications for this include asymmetrically shaped energy landscapes in nanofluidic cells for sorting nanoparticles based on their size (Fig. 4a). This so-called rocking Brownian motors can separate nanoparticles with only 1 nm difference in diameter^56,57^. Moreover, this technique has recently demonstrated the capability to place nanospheres and nanowires on top of prepatterned surfaces^58^.

Assembling Au nanorods by capillary forces in specifically shaped trap structures on PPA films with a precision of approximately ~10 nm is another example. A noteworthy benefit of PPA as a template is that it can be removed after particle assembly by heating the sample, leaving only the nanorods on the substrate^55^.

Grayscale t-SPL is also relevant for biophysics and tissue engineering. A recent study used t-SPL to pattern PPA so it mimicked the 3D topography of natural bovine tendon sections. Human mesenchymal stem cells adhered, spread and proliferated on these PPA nanostructures. This experiment demonstrates the capabilities of t-SPL and PPA for the study of cell–matrix interactions, including rapid prototyping of biocompatible scaffolds^59^.

#### Fabrication of templates and master molds

Fabrication of stamps for nanoimprint lithography (NIL) is an industrially relevant application of grayscale t-SPL. It combines the advantages of t-SPL to create complex grayscale topographies with nanoscale precision, such as holograms (Fig. 4b at the top) with NIL to provide the high throughput required for scalable industrial manufacturing^31^. Masters made of t-SPL patterned PPA are compatible with several commercial NIL replication processes like substrate-conformal imprint lithography (SCIL) or SmartNIL^TM^. Soft and transparent working stamps can be directly replicated by pouring the corresponding precursor solution onto the structured PPA. After curing, the replicated stamp can be used directly for the actual UV NIL process. Alternatively, the grayscale PPA structures can be etched into conventional master materials like silicon. The SEM images in Fig. 4b (bottom) show a grayscale sinusoidal grating in a transparent working stamp replicated from a silicon master and the imprint into a commercial glass-like material^31^.

Direct electroplating of metals on top of PPA is also possible. A Ni–Pd master prepared that way served as a shim for injection molding for high-throughput replication of grayscale nanopatterns into polymers, i.e., PMMA^60^.

#### Etch transfer of grayscale nanostructures from PPA into other materials

Grayscale patterns can be transferred by dry etching from PPA into other materials without significant loss of resolution. In one example, multilevel indent patterns in PPA were successfully transferred into silicon-on-insulator (SOI) substrates by reactive ion etching (RIE), which serve as hierarchical archival long-term data storage^61^.

Another example is a computer-generated hologram with eight individual levels (8 nm height difference), written in a single patterning step^31^. The 64 nm deep pattern in PPA was amplified to 700 nm in Si using RIE, which corresponds to a PPA/Si etch selectivity of ~11.

The capability of t-SPL to create grayscale structures makes it interesting to create high-quality optical elements. As an example, 30 nm deep Gaussian-shaped twin microcavities patterned by t-SPL into PPA, and subsequently etched into SiO_2_ were embedded in a distributed Bragg reflector stack (Fig. 4c). The precise Gaussian shape of the microcavities reduced losses and increased the quality factor up to 100×, as compared to conventionally used 2D mesa-shaped cavities. The coupling of twin microcavities can be tuned by varying the distance between them, thereby creating photonic molecules^54^.

t-SPL nanopatterns etched into thin Si_3_N_4_ membranes can produce phase masks for electron beam and X-ray optics. Such phase masks, for example, generated high-quality Vortex and Bessel electron beams in a transmission electron microscope (TEM)^62,63^ or 3D Fresnel zone plates for X-ray diffraction optics^64^.

Advanced memristor devices with vertical electrodes were shown to benefit from the conical shape of a single heated tip indent in the resist. Such indents in PPA on a pre-patterned substrate were etched into the SiO_2_ layer below the resist. After subsequent processing steps, a sharp tip-shaped Ag electrode embedded in a SiO_2_ matrix was obtained, which is separated by only 1 nm from a Pt electrode (see Fig. 4d). The resulting memristive device forms an atomistic metal filament between the electrodes allowing reliable switching at low voltages of 100 mV with operation speeds in the nanosecond range^65^. Such sharp 3D top contacts are increasingly relevant for many nanoelectronic devices.

#### High-aspect ratio etching with a transfer layer

The conical shape of the t-SPL tip and its short length of ~700 nm (typical commercial t-SPL cantilever) as well as the maximum electrostatic deflection of the cantilevers pose a limit to the achievable practical patterning depth. Patterns deeper than 200 nm tend to be less accurate and the thermal removal becomes unreliable since more material must be sublimated. Additionally, the high lateral resolution is lost due to the conical shape of the tip. However, some applications such as photonic structures require deeper nanostructures.

The etch depth in the substrate can be increased by adding a hard mask between PPA and the substrate which vertically amplifies the shallow patterns (Fig. 3d). As an example, shallow patterns in PPA (~30 nm) were amplified by a factor of 100 into silicon by the use of a 100 nm thick SiO_2_ hard mask. The t-SPL patterns were first transferred from PPA into the SiO_2_ hard mask by He/H_2_/C_3_F_8_ RIE and subsequently by SF_6_/C_4_F_8_ deep RIE from SiO_2_ into silicon, which resulted in structures as deep as 4 μm^66^. A potentially unwanted increase in roughness can be mitigated by an ion beam smoothening process after etching^67^.

#### Bilayer lift-off for advanced electronic devices

Lift-off processes are used to selectively deposit a material, usually a metal, onto a substrate by covering some areas during evaporation with a mask that is removed thereafter. A requirement for this process is an undercut in the resist layer in order to physically separate the deposited material on the substrate from the material on top of the mask. Implementing a lift-off process for t-SPL is straightforward: With two layers, the top layer is thermally removed by the hot tip followed by a chemical wet-etch of the bottom layer to create openings through both layers down to the substrate surface (Fig. 3e). The top layer is typically PPA and for the bottom layer any compatible lift-off resist can be used, e.g., polymethylglutarimide (PMGI). The bilayer lift-off is well suited for fabricating electrodes, in particular on 1D or 2D materials in combination with markerless overlay for source, drain and gate contacts.

In a recent work, this approach was successfully applied to fabricate single-layer MoS_2_ and WSe_2_ top-gated and back-gated field-effect transistors (FETs). Noteworthy, monolayers that are less than 1 nm thick and buried under >200 nm thick resist layers could still be detected by imaging with the cold tip for markerless overlay of the metal electrodes. Resulting metal contacts showed vanishing Schottky barriers and had record performances with an on-off ratio up to ten orders of magnitude and sub-threshold swings of 64 mV/dec for MoS_2_ transistors (Fig. 4e). In contrast, EBL-based fabrication is confirmed to induce higher Schottky barriers of the metal contacts, thereby limiting proper device performance^68^.

The feature density in the bi-layer lift-off process is limited by the isotropic wet etch step but gaps smaller than 20 nm and lines as thin as 20–70 nm are possible. To date, several variations of the bi-layer lift-off process have been developed and can be further adjusted for sample requirements such as hydrophobic or hydrophilic surfaces, water-free developer and low soft-baking temperature^69^.

#### Removal of resist to open functional surfaces below

Local removal of PPA by t-SPL can also be used to selectively uncover a functional material below the resist as illustrated in Fig. 3f. This is for example useful for selective assembly of molecules or particles, as depicted in Fig. 4f. This system consisted of a PPA layer on top of a cross-linked polymer with protected amine groups. During t-SPL, the thin PPA resist layer was removed down to the underlying material. The heat from the tip triggered simultaneously the deprotection of the amine groups in the underlying layer. While PPA is negatively charged in aqueous solution, the exposed amine groups become protonated. These patterns were used to study the interplay between diffusion and surface reactions in the assembly process of negatively charged particles in dilute solutions which are relevant for modern sensor applications^70–72^.

A similar strategy was used to selectively activate a brush polymer for directed self-assembly of PS-b-PMMA block co-polymers. Directed self-assembly of block co-polymers is a promising route to increase the resolution of industrial semiconductor device manufacturing by aligning the polymers between lithographically defined features to multiply the feature density. Line patterns in a 3.5 nm thick PPA layer on top of a brush polymer were created by t-SPL. The uncovered brush polymer layer was then activated by a short oxygen plasma treatment and the remaining PPA was removed, which resulted in a surface having areas that exhibit two distinctly different polarities. Subsequent directed self-assembly of PS-b-PMMA block co-polymers on this surface resulted in well-defined structures with a half-pitch of 11.7 nm over tens of square micrometers^73^.

#### Ultra high-resolution multi-layer transfer

Long and small resist features become mechanically unstable and are likely to collapse with decreasing vertical size and increasing aspect ratio. This is one reason why high-resolution lithography in semiconductor industry uses thinner resist layers for smaller structures (<20 nm resist thickness for some EUV processes). However, these thin resists with high-resolution features often miss the requirements for deep and anisotropic etch transfer into materials like Si, as required for FinFETs for example. The main strategy for high-resolution transfer with t-SPL is to use a 2–3 nm thin inorganic hard mask (HM) and an organic transfer layer (OTL) below the <10 nm thick PPA resist layer as illustrated in Fig. 3g, h. The shallow PPA pattern is transferred first into inorganic HM using RIE. The OTL is then etched by an anisotropic O_2_ plasma which has a high selectivity to the inorganic HM. A 2 nm thick PMMA layer can be additionally used between PPA and the HM to reduce tip wear^29^. The quality and the thickness of the inorganic HM plays a very important role for the transfer process. SiO_2_ was found to be an ideal HM in combination with PPA. The SiO_2_ HM layer can be deposited by sputtering^74^, but experimentally it was found that evaporated films showed better results. For a 2.5 nm thick SiO_2_ layer deposited by e-beam evaporation, 7 nm wide straight lines at a half-pitch of 14 nm have been obtained^29^ (see Fig. 4g left). More recently, it was demonstrated that a high quality, 3 nm thin SiO_2_ HM layer can be created also by spin-coating of a spin-on-glass resist, which simplifies the process^69^.

To increase the etch resistance of PPA, it can be modified after t-SPL patterning to become a highly selective etch mask itself. In a so-called sequential infiltration synthesis (SIS) process, the patterned PPA layer is infiltrated by a tri-methyl-aluminum precursor using atomic layer deposition (ALD) and converted into Al_2_O_3_. The resulting inorganic hard mask serves as an etch mask for other layers. Smooth vertical Si fins with a width of 10 nm and as deep as 70 nm were obtained after dry etching^75^. In Fig. 4g on the right side, a TEM image of such a Si fin is shown.

In combination with PMMA as an OTL, a lift-off process was developed, where the undercut is created by RIE from the HM layer into PMMA. This anisotropic etch does not limit the resolution as opposed to the isotropic etching in the bi-layer lift-off process. This allowed, for example, fabrication of high-resolution plasmonic structures and top-gate electrodes on InAs nanowires^76^. The resulting InAs transistor shows switching close to the theoretical limit of 60 mV/dec (see Fig. 4h) and demonstrated that the electronic properties are not harmed during t-SPL^77^.

To benefit from the high resolution of t-SPL and the fast patterning speed of direct-write laser lithography, a mix-and-match approach can be used as depicted in Fig. 4i. In this example, single-electron transistors based on point-contact tunnel junctions have been fabricated in silicon^43^. In a first step, the high-resolution patterns were written into PPA by t-SPL. In a second step within the same tool, the large low-resolution patterns were added to the PPA resist by thermal evaporation with a focused laser. The PPA patterns were transferred into Si using the previously described process involving a SiO_2_ HM and an OTL to etch the required high resolution features. This example demonstrated the first hybrid micro–nano direct-write system, where a laser was integrated into the t-SPL setup.

## Material conversion

**Fig. 5:**
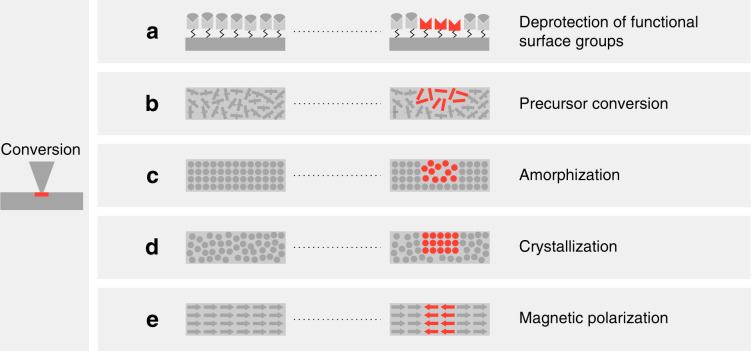
Overview of thermal conversion processes. **a** Deprotection of functional surface groups^32,33,78–82,84,85^. **b** Conversion of a functional material from a precursor^42,45,86–92,94,95^. **c** Amorphization by short heating and fast subsequent quenching to obtain a less ordered phase^96,97^. **d** Local crystallization of an amorphous material^21,98–102^. **e** Heat-assisted local alignment of magnetic dipoles^104–107^

**Fig. 6:**
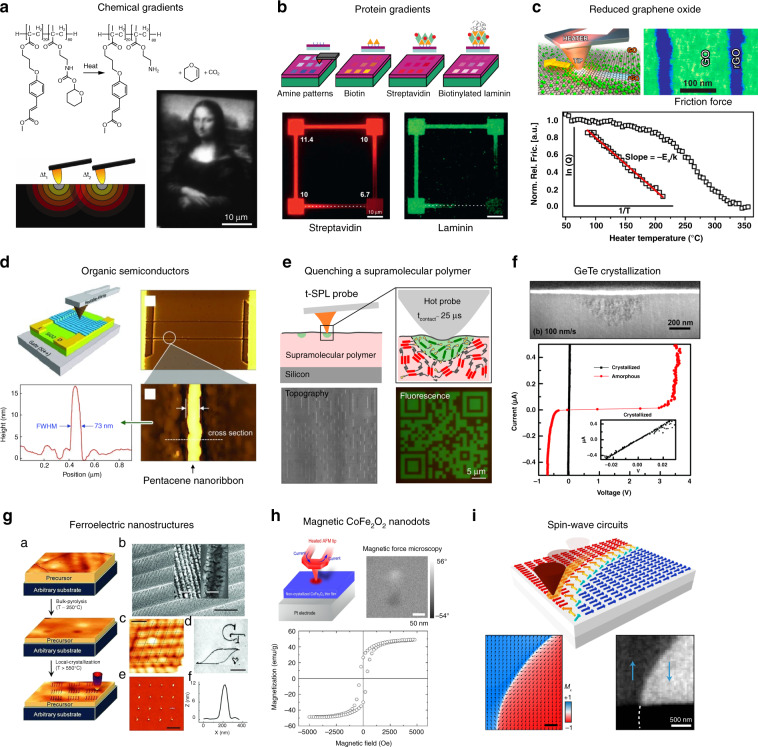
Examples of applications achieved by material conversion using t-SPL. **a** Fabrication of nanoscale chemical gradients through local deprotection of THP-capped functional amine groups with a heated probe. Adapted with permission from ref. ^33^, Copyright 2013 ACS. **b** Fabrication of protein gradients via thermal deprotection of amine groups and subsequent functionalization with proteins. Reproduced with permission from ref. ^82^, Copyright 2016 IOP Publishing. **c** Modification of the surface chemistry of graphene oxide with a thermal probe to study reaction kinetics on the nanoscale. Adapted with permission from ref. ^45^, Copyright 2017 ACS. **d** Fabrication of pentacene nanowires from a precursor material by thermally activated conversion reaction Reprinted with permission from ref. ^92^, Copyright 2013 John Wiley & Sons. **e** Thermal quenching of a thermochromic supramolecular polymer to obtain a fluorescence contrast via disaggregation of excimer-forming moieties. Reproduced with permission from ref. ^96^, Copyright 2017 ACS. **f** Local crystallization of a GeTe phase-change material with a heated probe to create electrically-conducting nanostructures. Republished with permission from ref. ^98^, Copyright 2017 RSC Pub. **g** Direct fabrication of ferroelectric PZT nanostructures by local crystallization of a pyrolyzed amorphous sol–gel precursor. Reproduced with permission from ref. ^99^, Copyright 2011 John Wiley & Sons. **h** Fabrication of nanoscale magnetic vortices by local crystallization of CoFe_2_O_2_ films. Reprinted with permission from ref. ^101^, Copyright 2018 Elsevier. **i** Reconfigurable magnetic nanostructures via t-SPL patterning by application of an external magnetic field which re-orients the thermally exposed area for the fabrication of spin-wave circuits. Adapted under CC BY 4.0 License from ref. ^106^, Copyright 2018 Springer Nature

As mentioned in the introduction, heat can drive a wide range of processes in almost any material and is therefore interesting as a stimulus for patterning. Figure 5 provides an overview of modifications achieved by t-SPL, where highly localized heating is used to induce patterns of chemical and/or physical modifications directly in a material. Chemical modifications, on the one hand, involve the creation or break-up of bonds in the material (Fig. 5 a, b). Physical modifications, on the other hand, involve the heat-induced rearrangement of atoms to facilitate, for example, crystallization or alignment of dipoles (Fig. 5c–e). The appeal of local thermal conversion lies in the ability to modify intrinsic material properties, such as the surface chemistry or the electrical conductivity, at the nanometer scale in a single patterning step. Figure 6 shows an overview of examples where local heating was used to induce modifications to produce a distinct application-specific contrast. The following sections discuss heat-induced chemical and physical conversion with thermal probes.

### Chemical conversion

#### Deprotection of functional groups

Control of the surface chemistry at the micrometer and nanometer scale is important due to the quest for highly selective biosensors with tailored surface properties. t-SPL offers a unique way to selectively modify a sample’s surface chemistry, for instance to locally uncover functional groups (e.g., amine or hydroxyl groups) that have been previously covered with a protecting group (e.g., tetrahydropyran (THP) or tertiary butyl ester groups). Ideally, the thermal deprotection reaction is fast and occurs in a narrow temperature window that does not overlap with other thermally-triggered reactions such as degradation. In addition, the polymer film should be mechanically stable beyond the temperature at which the deprotection reaction occurs. In other words, the polymer must have a high glass transition temperature in order to avoid softening of the film due to possible tip contamination and undesired material flow.

Variations of methacrylate block-copolymers, composed of a photocrosslinkable moiety and a THP-protected functional moiety have been identified as promising candidates^78–81^. Mechanically stable polymer thin films are obtained by spin-coating the solution followed by UV crosslinking. Figure 6a shows the thermal deprotection reaction of THP from a functional amine group. Typical probe temperatures at which the deprotection takes place range between 160 and 240 °C at a patterning speed between 10 and 200 µm/s. A record patterning speed up to 2 mm/s has been demonstrated for such conversions^79^.

A compelling benefit of the serial writing process of t-SPL is that the temperature and tip-sample contact duration can be locally set, which permits precise control of the fraction of functional and protected sites^32,33^. Figure 6a shows a fluorescently labeled replication of Leonardo da Vinci’s “Mona Lisa” image, which was fabricated by setting the temperature at the tip-sample contact according to the grayscale value of the image, modulating the fraction of the deprotected functional groups. With the same technique, gradients of streptavidin and laminin obtained by sequential functionalization were obtained as shown in Fig. 6b^82^.

Another advantage of chemical deprotection by t-SPL is the possibility to create multiplexed nanopatterns that can be fabricated by the following approach. In the first step, patterns of deprotected primary amines are written with a heated probe and then thiolated by wet immersion. In the second step, a different set of patterns is created on the same sample using the same thermal probe, which results in areas that are amine-functionalized side by side to the thiolated areas^79^. Chemically deprotected groups were used to locally bind proteins, antibodies, DNA^79^ or more recently also enzymes at a resolution as high as 10 nm^80,81^.

In yet another study, self-assembled monolayers were patterned to selectively bind gold nanoparticles and carboxylic acid terminated molecules^83^. Similar to THP, tertiary butyl ester groups have been successfully used to protect functional groups followed by localized deprotection with heated probe^84,85^.

#### Conversion from a precursor material

Many chemical reactions can be activated by heat. A common strategy to obtain nanostructures of the desired material and property is to use a precursor material that is locally converted by the heated tip.

One example is the reduction of graphene oxide (GO) flakes and films to obtain electrically conducting reduced graphene oxide (rGO). Thermal patterning with the heated probe locally reduces the oxygen content of the GO, increasing the electrical conductivity by four orders of magnitude^42,86^.

In another example, GO was thermally reduced to rGO with a heated probe to investigate the kinetics of the reaction^45^. Experimentally, the reaction kinetics was determined by correlating the probe-sample friction with the oxygen concentration of the sample (Fig. 6c). Thermochemical oxidation of graphene is also possible when it is heated with a thermal scanning probe in a humid atmosphere^87^.

Not only low-dimensional materials can be chemically converted by t-SPL, but also thin films up to a certain depth, which requires the capacity of the thermal probe to heat a larger volume. In contrast to thermal deprotection of functional groups at the surface, the main interest here is to convert a well-defined volume of the precursor material, mainly to modify a specific material property, such as its electrical conductivity. Ideally, the thermochemical conversion reaction takes place in a narrow temperature range and at time scales that are sufficiently short to pattern at a reasonable speed.

The synthesis of poly(p-phenylene vinylene) (PPV) from a sulfonium salt precursor via thermal elimination is a widely studied thermally triggered reaction. PPV is interesting as an organic material due to its electroluminescence and electrical conductance when doped. Typically, 15–100 nm thick precursor films are locally heated with a t-SPL probe to temperatures between 240 and 350 °C at scanning speeds between 20 and 80 µm/s^42,88–91^. By preheating the substrate to 85 °C, the patterning speed could be increased above 1 mm/s^88^. During thermal exposure, only a fraction of the precursor molecules is converted to PPV, which is still sufficient to induce a solubility change in the thermally exposed material. After developing, post-baking of the remaining PPV nanostructures at 200 °C completed the conversion^88,89,91^.

Another well-studied thermally triggered conversion is the retro Diels-Alder reaction from 13,6-N-sulfinylacetamidopentacene to pentacene, a semiconducting organic material. Pentacene nanoribbons (70–100 nm wide and 20 nm thick) were obtained by t-SPL at a patterning speed between 0.1 and 32 µm/s (Fig. 6d). The highly anisotropic characteristics of these nanoribbons are attributed to their alignment with the tip-scan direction. Incorporated in bottom gate field-effect transistors, the converted pentacene exhibited p-channel behavior and hole mobility up to 10^−3^ cm^2^/Vs^88,92^. This value is two orders of magnitude lower than comparable solution-processed devices^93^, which leaves room for improvement in the fabrication of organic semiconductor devices by t-SPL. Another versatile approach to induce chemical reactions by t-SPL was demonstrated using 1,3-cycloaddition of diazides and diyenes to form poly(arylenetriazolylene)—so-called “Click chemistry”^94^.

In commercial negative photoresists, ultraviolet (UV) light-induced cross-linking is a common mechanism to create a solubility contrast between exposed and unexposed areas. Cross-linking of commercial photoresists (AZ 5214E) with heated probes produces nanostructures below the optical diffraction limit, which extends the repertoire of t-SPL resists to negative tone resist. Prior to thermal pattering, the entire photoresist is exposed to UV light. Then the contrast is obtained by local resist baking with the heated probe, which induces cross-linking and renders the exposed material insoluble^87,95^. Note that cross-linking of resist by t-SPL can be challenging due to tip contaminations and the fact that thermal exposure down to the substrate is difficult. Pattern inversion with a positive resist (e.g., removal of PPA) involving a lift-off and subsequent etching process is more appropriate for many applications requiring negative patterning (e.g., due to the required patterning time).

### Physical conversion

Phase changes in many materials are driven by heat, permitting the material to reach a more energetically favorable state. The possibility to control such changes locally enables unique applications that are sometimes not feasible with other methods.

#### Local quenching

The fast heating and cooling rates attainable in t-SPL enable local quenching at the nanometer scale. Due to the small volumes heated in t-SPL, after removal of the probe, the heat quickly dissipates into the surrounding bulk material. If recrystallization takes longer than cooling of the exposed volume the material can be kinetically trapped.

This was successfully demonstrated by using a thermochromic aggregate-forming supramolecular polymer as illustrated in Fig. 6e. Melt-processed films of the material exhibit a red fluorescence due to aggregation of excimer-forming fluorescent moieties. Upon heating and quenching the material with a thermal probe, the excimer-forming moieties disaggregate, which results in a distinct emission color shift from red to green^96^. Similarly, water-insoluble annealed silk fibroin thin films were patterned by t-SPL, inducing a water solubility contrast between exposed and unexposed material. Here, short heat pulses and localized heating are used to melt β-sheet crystallites in the water-insoluble silk fibroin phase, making the exposed material water-soluble. Development of the material in water resulted in a distinct topography contrast^97^.

#### Local crystallization

Heat from a thermal probe can also induce local crystallization of an amorphous material by activating diffusion. For crystallization to occur, sufficient heat needs to be transferred from the tip to the sample such that the temperature is sufficiently high for considerable diffusion to take place.

In one of the earliest t-SPL experiments, crystalline nanostructures with a lateral resolution below 40 nm were created in amorphous Ge_2_Sb_2_Te_5_ chalcogenide films using a laser-heated AFM cantilever^21^. More recently, tip-induced crystallization of amorphous GeTe films was achieved at tip-scanning speeds between 100 nm/s and 1 µm/s^98^. Due to the density change of the crystallized area, the modification could be detected by AFM surface scans. TEM cross-section images confirmed the extent of the crystallized area as shown in Fig. 6f. As expected, electrical characterization of the patterned nanostructures showed that the crystallized nanostructures became electrically conducting, in contrast to the non-conducting amorphous GeTe^98^.

Arbitrarily-shaped ferroelectric nanostructures were directly fabricated on plastic, silicon or glass substrates in a three step process: (i) deposition of sol–gel precursor of Pb(Zr,Ti)O_3_ or PbTiO_3_ on substrate, (ii) bulk pyrolysis at 250–300 °C and (iii) subsequent local crystallization of the 180–400 nm thick pyrolyzed film with a heated tip as shown in Fig. 6g. The 10–80 nm wide nanostructures exhibited reversibly switchable piezoelectric hysteresis behavior^99^. More recently, a similar sol–gel-based thermal conversion of ferroelectric BiFeO_3_^100^ and ferromagnetic CoFeO_3_^101^ nanostructures have been reported as illustrated in Fig. 6h. These sol–gel precursor-based local annealing fabrication techniques have in common that the tip-sample contact duration is long (between 0.6 and 150 s) and high tip-sample contact temperatures are required (550–700 °C). It can be expected that the exposure temperature could be reduced by substrate preheating or better thermal isolation of the sample to reach higher contact temperatures for inorganic materials. For organic materials lower temperatures are required. As an example, ferroelectric crystalline poly-(vinylidene fluoride-trifluoroethylene) (PVDF-TrFE) nanodots were obtained by t-SPL at temperatures as low as 160–200 °C during a 2–10 s long annealing with the probe^102^.

While these examples demonstrate the feasibility of local crystallization with a heated atomic force tip to induce phase changes accompanied by material property modifications such as electrical conductivity, piezoelectricity, or magnetic polarizability, currently the annealing times in the order of milliseconds to seconds per indent are too long for applications beyond experimental studies and more optimization of materials and processes is necessary.

#### Local heat-assisted magnetic domain switching

A heated tip has also been used to create nanoscale magnetic domains with arbitrary shape and direction of magnetization. This is possible by locally heating a sample composed of multilayer stacks of ferromagnetic and antiferromagnetic films while simultaneously applying an external magnetic field^103^. The resulting polarized domains are stable at room temperature but can be simply erased by heating above 160 °C without an external magnetic field. This unique capability to pattern nanoscale domains, and in particular, to purposely design individual domain walls, is very interesting for a range of applications. As an example, this technique was successfully applied to create and control vortex/antivortex pairs and Bloch lines, to guide spin waves (Fig. 6i), and to create previously unknown optics-inspired magnonic circuits, e.g., nanoantennae for spin waves^103–107^.

## Material addition

**Fig. 7:**
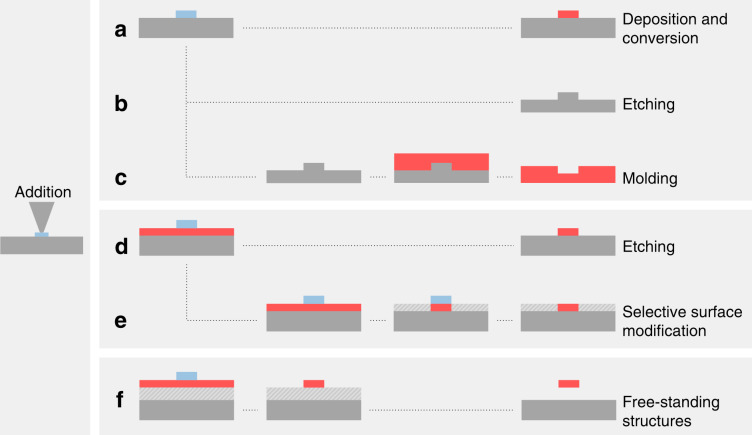
Overview of relevant nanofabrication processes after addition of material to the sample from a heated tip. **a** Direct deposition of a molten material^108,111–116,127^ or a loaded carrier matrix (e.g., polymer containing nanoparticles), which can be removed after the transfer^117^. **b** Direct deposition of a resist for dry etching into the substrate material^118,121^. **c** The etched structure can be used as a master for molding^118^. **d** Direct deposition of etch-mask nanostructures for a solvent free pattern transfer^119,120^. **e** Local functionalization of a low-dimensional material by selectively protecting certain areas with a nanoscale mask^110^. **f** Fabrication of free-standing nanostructures by dry etching into a first layer and subsequently removing of the sacrificial layer below^122^

Besides material removal and material conversion as described above, t-SPL is also capable of adding material to a substrate. One common method uses a heated tip that is loaded with a low-melting-temperature material. When the tip is heated and brought into contact with a substrate, a flux of the melt is established across the tip-sample contact. Scanning the tip over the substrate creates arbitrary patterns that can be used for subsequent processing as shown in Fig. 7. Another method performs local chemical vapor deposition (CVD). Instead of heating the entire sample as in conventional CVD processes, the reaction and deposition takes place only in a confined region heated by the tip, as described below.

### Melt transfer

In thermal dip-pen nanolithography, the target material—typically a solid at room temperature—is loaded onto the cantilever prior to patterning. To transfer material from the tip to the substrate, the tip is brought into contact with the substrate and heated above the melting temperature of the loaded material. This is typically done with a resistive heater element in the cantilever, which melts the loaded material and establishes a mass flux from the tip apex to the sample^108^. To write arbitrary patterns, the tip is moved with respect to the substrate. The deposition flow rate can be controlled by adjusting the cantilever temperature, thereby changing the viscosity of the melt, and also by adjusting the scan speed^109^. The melt flow from the tip to the substrate is governed by shear or surface tension forces. The capillary number Ca = *μV*/*γ* gives the relative importance of the viscous stress, determined by the material viscosity *μ* and the probe speed *V* to the surface tension stress, determined by the polymer surface tension *γ*^108^. As an example, for polyethylene (PE) melt, it was found that surface capillary forces dominate the polymer flow over shear forces between tip and substrate^108^. The tip temperature can range from 100 to 200 °C for polymers and from 250 to 800 °C for metals depending on the material’s melting temperature. Typical patterning speeds are between 0.1 and 5 µm/s, though some experiments have demonstrated that patterning at up to 40 µm/s is possible^110^. If necessary, the surface can be scanned with the cold tip prior or after patterning to image the topography for alignment purposes.

A key advantage of this additive technique is that polymeric nanostructures can be deposited onto almost any material without the need for solvents or developers. The deposited nanostructures can be further transferred into other materials via standard processes such as etching and molding or can serve as a protection layer for selective-surface modification, as summarized in Fig. 7.

#### Direct deposition of functional materials

Deposition of octadecylphosphonic acid (OPA) that forms self-assembled monolayers on various substrates such as mica, metals and metal oxides were among the first experiments where material was transferred from a heated tip to the substrate^111^. OPA was loaded onto the cantilever by thermal evaporation. Only when the cantilever was heated above the melting temperature of OPA (99 °C) material was transferred.

**Fig. 8:**
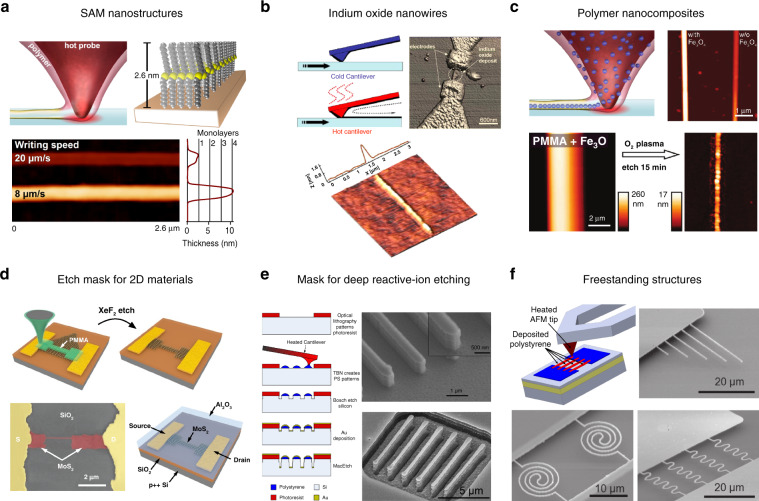
Examples of nanostructures fabricated by addition of material with a thermal scanning probe. **a** Heat-assisted deposition of PDDT self-assembled nanostructures from a preloaded tip with control of the layer thickness by the probe speed. Reproduced under CC BY 2.0 License from ref. ^113^. **b** Deposition of molten indium from a heated tip. Reprinted with the permission from ref. ^116^, Copyright 2006 AIP Publishing. **c** Deposition of a polymer loaded with nanoparticles. Subsequent oxygen plasma treatment removes polymer matrix and leaves solely the nanoparticles on the surface. Adapted with permission from ref. ^117^, Copyright 2010 ACS. **d** Direct deposition of PMMA nanostructures as a mask for XeF_2_ plasma etching of MoS_2_ flakes. Adapted with permission from ref. ^120^, Copyright 2019 ACS. **e** Deposition of PS as an etch mask for Bosch etching with subsequent metal-assisted etching. Reprinted with permission from ref. ^121^, Copyright 2013 AVS. **f** Fabrication of free-standing nanostructures on a SOI wafer by direct deposition of a PS etching mask, dry etching and subsequent wet etching. Reproduced with permission from ref. ^122^, Copyright 2014 IOP Publishing

In a similar approach, self-assembled monolayers of poly(4-dodecylthiophene) (PDDT) were achieved^112^. The number of monolayers can be precisely controlled by the scanning speed of the heated probe^113^ as shown in Fig. 8a. Scanning the sample with the cold tip prior to patterning in order to image the surface permitted to precisely place the PDDT between two electrodes separated by a gap of 1 μm^114^.

Another example of thermal probe-assisted deposition is poly(N-isopropylacrylamide) (PNiPAAm) nanostructures on epoxy-terminated substrates and was used for reversible binding of the proteins neutravidine and bovine serum albumin^115^.

Direct deposition of nanostructures is not limited to organic materials; low melting temperature metals can also be successfully transferred. In one example, indium (In) was loaded onto the cantilever by scanning an In substrate at a high tip temperature and contact force of ~1030 °C and 500 nN, respectively. Indium melt was then transferred onto another surface by heating the cantilever in contact with the sample and scanning over the substrate as shown in Fig. 8b^116^. It was found that the width of the deposited line depends on the tip load, cantilever temperature and the deposition speed. Elemental analysis of the nanostructures revealed that indium oxide was obtained due to the strong tendency of In to oxidize. This technique could be potentially interesting for the deposition of arbitrarily-shaped liquid metal nanostructures.

#### Polymer inks as a carrier matrix

Another interesting application of this technique is the deposition of nanoparticles in a polymer ink. The polymer acts as a carrier matrix for the coating of the tip and the subsequent deposition of the ink from the heated tip onto the sample. Subsequent oxygen plasma treatment of the composite structures removes the polymer and leaves the nanoparticles on the substrate as schematically shown in Fig. 8c. Various nanoparticles and quantum dots have been locally deposited this way^117^.

#### Direct etching and molding

The transferred material can also be used as a direct-write mask for pattern transfer. A commonly used material is polystyrene (PS). Arbitrarily-shaped PS nanostructures were directly transferred into silicon by means of reactive ion etching, whereby the PS served as an etch mask with an etch selectivity as high as 1:80. From the resulting silicon master, 10 μm long nanofluidic channels with a cross sectional area of 300 × 500 nm have been replicated into polydimethylsiloxane (PDMS)^118^.

#### A mask for etching and surface modification

Chemically and geometrically isolated graphene nanoribbons were fabricated by depositing PS nanowires onto graphene sheets. The polymer served as a mask for subsequent fluorination and oxygen plasma etching, which convered the exposed graphene into an insulator and etched the graphene away, respectively^110,119^. Direct deposition of the polymer with a heated tip onto graphene is advantageous in comparison to e-beam because no electrons that irreversibly damage the material are involved and no resist residues are left after development. Recently, transistors with 30 nm wide nanoribbons of MoS_2_ were fabricated by using t-SPL to deposit a PMMA resist onto MoS_2_, which served as mask for subsequent XeF_2_ dry etching as illustrated in Fig. 8d^120^.

Not limited only to the etching of low-dimensional materials, tip-deposited PS nanostructures have been successfully used as a mask for deep reactive ion etching (DRIE), also known as "Bosch" process and metal-assisted chemical etching of silicon to obtain high aspect ratio nanostructures (Fig. 8e)^121^. Arbitrarily-shaped free-standing silicon nano-resonators have been fabricated on a silicon-on-insulator substrate, as shown in Fig. 8f^122^.

At the moment, a major challenge of this technique is to control the flow of molten material from the tip to the substrate. Ongoing efforts address this issue by designing advanced cantilevers with improved mass flow control^123^.

### Addition from the gas phase

The idea to use t-SPL for local chemical vapor deposition (CVD) is intriguing since it would permit depositing a wide variety of high-quality materials in arbitrary shapes at the nanoscale without additional processing steps. So far, only a few experiments have been published in literature. Cu and CuO nanostructures were fabricated by using sublimated copper acetylacetonate in a low pressure Ar/O_2_ atmosphere. The copper acetylacetonate reacted to Cu or CuO on the locations of a Si substrate that were heated by a tip to a temperature of around 300 °C^124,125^. While not discussed in literature, one would expect that the CVD reaction would not only take place on the substrate but also on the heated tip and hence contaminate it over time. Another interesting mechanism to be explored would be localized doping via diffusion of atoms from the gas phase to where the tip is heating the substrate.

## Conclusion and outlook

We have reviewed the state of the art in t-SPL through the investigation of three categories: removal, conversion, and addition, all with a particular focus on materials and processes. It was shown that nanoscale heating with a scanning probe enables a multitude of ways to create high-resolution patterns on surfaces for a wide diversity of relevant applications.

Our review finds that heat-induced removal of resist is the most commonly used category of t-SPL. The main reason for this is that the created topographical nanopatterns are efficiently written, can be immediately inspected and transferred into almost any material of choice by means of standard microfabrication processes such as etching and thin film deposition. Therefore, the capabilities of t-SPL for removal of resist are comparable to well-established EBL.

The main advantage of t-SPL, however, lies in its unique capabilities. For example, 3D (grayscale) patterning can be achieved with unprecedented accuracy using t-SPL and has already enabled new nanodevices. The unique marker-less overlay capability of t-SPL simplifies device fabrication, in particular when randomly positioned features, like 2D material flakes or dispersed nanowires, are involved. Finally, the lack of charged particles in t-SPL enables the fabrication of superior nanodevices with materials and architectures that would otherwise suffer from damage typically caused by charged particle-based nanolithography methods.

We have compiled a comprehensive list of materials that have been demonstrated to undergo physical or chemical conversion upon heating through t-SPL. Many examples for local material conversion have already been published in literature, but many more possibilities for material conversions have not yet been discovered or tested due to the relatively small user base and young age of commercially available t-SPL tools. We would like to emphasize that t-SPL material conversion is not only interesting because of the small dimensions that can be patterned. The extremely high heating and cooling rates as well as wide range of achievable tip pressures can lead to novel types of material modifications that are not feasible at macroscopic dimensions.

The same is certainly true for material addition by t-SPL. Several publications about the deposition of solid inks from a thermal probe have been reviewed here, but the local deposition of material from the gas phase appears almost completely unexplored and leaves plenty of room for new discoveries.

Challenges for every intended t-SPL reaction include the difficulty to precisely predict the accurate temperature at the tip-sample contact, and the unknown reaction kinetics with the typical ultrahigh heating and cooling rates of t-SPL as well as the potentially high pressures under the tip. Further research is required to understand the heat transfer at the nanogap between tip and sample during thermal patterning. Ideally, the temperature of the thermally converted material should be directly monitored during patterning. It would be also interesting to better understand the transient heat transfer at the moment when the hot tip contacts the sample until it reaches steady state, and how this initial temperature change affects the reaction kinetics of the sample modification. Tip contamination and tip wear pose other challenges, which require further improvements in tip stability and development of resists that do not leave residues after thermal conversion^126^. Finally, the effect of pressure due to the nanoscale tip on the reaction kinetics is a topic that has not been well investigated except in the case of graphene oxide^45^.

On a more positive note, while accurate measurements and theoretical descriptions of the tip temperature remain insufficient, experimentally monitoring modifications of a material is possible due to the in situ AFM imaging. Modern t-SPL setups are flexible and relatively straightforward, so samples and materials can be quickly tested with varying temperatures, forces and heating rates. The unique asset of in situ imaging using the cold tip is that the surface modification process can be quickly optimized.

Today, t-SPL is still mainly used for research applications where it can provide additional advantages over conventional nanolithography methods, such as the cost of ownership, rapid prototyping, material choice and quality or direct 3D/grayscale patterning. First commercial t-SPL systems have been recently installed in industry; however, the main barrier for broader acceptance in both academic and industrial manufacturing remains the current limit in throughput.

To overcome this barrier, the technology is being pushed towards hybrid thermal lithography where the benefits of fast laser-writing and high-resolution t-SPL are combined in a single tool: large areas of a temperature-sensitive resist can be rapidly modified with a laser and the areas of the same sample that require high resolution can be patterned by t-SPL using the same resist^43,62^. The development of new t-SPL systems with multiple cantilevers that can be simultaneously actuated is also ongoing and proof-of-principle experiments with linear arrays of up to ten cantilevers have been demonstrated^42,62^. Multiple t-SPL scan heads and parallel arrays with thousands of cantilevers similar to IBM's Millipede device would enable industrial manufacturing of advanced 3D photomasks and large area stamps for nanoimprint lithography. The throughput of t-SPL that may be achieved with this parallelization will be still too low for high-volume industrial manufacturing required for today’s and tomorrow’s semiconductor chips. However, the rapid prototyping capabilities of t-SPL might become essential for the semiconductor industry in chip and process development. Furthermore, the various unique capabilities of t-SPL may also be applied in industrial direct-write manufacturing applications for devices that require modifications of only small critical areas. In the near future, we foresee a continuously growing impact of t-SPL with technological demonstrations of novel nanodevices and further scientific discoveries.

## Supplementary information

Supplementary Information
