# Supplementary material for: Thermal scanning probe lithography—a review
Source: Microsyst Nanoeng. 2020 Apr 6;6:21. doi: 10.1038/s41378-019-0124-8 (PMC8433166; doi:10.1038/s41378-019-0124-8)
Supplement: Supplementary file 1 — Supplementary Information [file 41378_2019_124_MOESM1_ESM.docx]

THERMAL SCANNING PROBE LITHOGRAPHY – A REVIEW

(Supplementary Information)

Samuel Tobias Howell^†^, Anya Grushina^‡^, Felix Holzner^‡^, Jürgen Brugger^†^

*^†^Microsystems Laboratory, Ecole Polytechnique Fédérale de Lausanne (EPFL), 1015 Lausanne, Switzerland*

*^‡^Swisslitho AG, Technoparkstrasse 1, 8005 Zürich, Switzerland*

## Tip-sample Contact Temperature

Most t-SPL cantilevers use a resistive heater that is located directly above the tip to generate heat. For doped silicon heaters, its temperature can be inferred by a fix-point calibration of the knee temperature at which the resistance is highest and can be calculated analytically^1^; or as recently shown to be more accurate (<25 K), by correlating the heater temperature with the dissipated power ^2^. The temperature at the tip-sample point of contact is far less straightforward to determine owing to the complex interplay of many factors such as the difficulty to measure or calculate the thermal boundary resistance, heat losses or the exact tip-apex shape. Here we discuss a simple resistive heater model to compute the tip-sample contact temperature that has been proposed in literature^3,4^. For simplicity, radiative and conductive heat transfer through the cantilever-surface gap are neglected. Based on the thermal circuit shown in Figure 2a in the main manuscript, the tip-sample contact temperature $T_{ts}$ can be calculated as follows:

$$T_{ts}=c\cdot\left( T-T_{0} \right)+T_{0} (1)$$

where $c={R_{sp}}/{(R_{tip}+R_{ts}+R_{sp})}$ is the heating efficiency that can be calculated from the tip thermal resistance $R_{tip}$, tip-sample boundary resistance $R_{ts}$, and the spreading resistance of the sample $R_{sp}$. $T$ and $T_{0}$ are the heater and sample temperature, respectively. Note that the thermal resistance model is only valid for steady state heat transfer (Fourier number $Fo=\frac{\alpha t}{L^{2}}\gg1$). For typical length scales $L$ (<100 nm), time scales $t$ (>1 µs) and diffusivities $\alpha$ (10^-7^ – 10^-5^ m^2^/s) as relevant in t-SPL this assumption is valid. As an example, Fourier numbers of >10 and >10^3^ are obtained for polymers and silicon, respectively.

The thermal resistance of a conical tip with an opening angle $\theta$ is inversely proportional to the thermal conductivity $\kappa_{t}$ of the tip material. However, due to the small diameter of the tip$d_{0}$ – on the order of the mean free path of phonons $\lambda$ – boundary phonon scattering considerably increases the thermal resistance as compared to the bulk material^3^:

$$R_{tip}\approx\frac{\lambda}{\pi\kappa_{t}\tan\left( \frac{\theta}{2} \right)d_{0}^{2}}. (2)$$

The tip thermal resistance for typical t-SPL tips with a tip apex radius of a few tens of nanometers is on the order of 10^6^-10^7^ K/W^3^.

The tip-sample contact resistance can be approximated by

$$R_{c}=\frac{{4R}_{b}}{\pi d_{eff}^{2}}, (3)$$

where $R_{b}$is the boundary resistance which ranges between $5\times{10}^{-9}-5\times{10}^{-8} \text{m}^{\text{2}}\text{K/W}$ for a tip-polymer system^4,5^ and $d_{eff}$ is the effective tip diameter which is dependent on the indentation depth. For small indentation depths z the effective tip diameter of a spherical tip can be approximated ^4^ as $d_{eff}\left( z \right)=2\sqrt{d_{0}z}$ while for large indentation depths of a conical tip^6^ with an opening angle $\theta, d_{eff}\left( z \right)=2\frac{\sqrt{\sin\left( \frac{\theta}{2} \right)}}{\cos\left( \frac{\theta}{2} \right)}z$. For a conical tip with a spherical tip apex $d_{eff}\left( z \right)=2\frac{\sqrt{\sin\left( \frac{\theta}{2} \right)}}{\cos\left( \frac{\theta}{2} \right)}\sqrt{z^{2}\left[ 1+\frac{d_{0}}{z}\left( \frac{1}{\sin\left( \frac{\theta}{2} \right)}-1 \right) \right]}$.

The heat transmitted through the tip-sample contact radially diffuses away through the substrate material with a spreading resistance $R_{sp}=1/(2\kappa_{s} d_{eff})$ inversely proportional to the substrate thermal conductivity $\kappa_{s}$. For a substrate covered with a thin film of a thickness $t$ and a thermal conductivity $\kappa_{f}$, the thermal spreading resistance can be approximated (2 < t/d < ∞) ^7^ by

$$R_{sp}=\frac{1}{2\kappa_{f}d_{eff}}-\frac{1}{2\pi\kappa_{f}t}\ln\left( \frac{2}{1+\kappa_{f}/\kappa_{s}} \right). (4)$$

Values for the thermal spreading resistance range from 10^8^ K/W for polymers to 10^5^ K/W for silicon^3^.

Heating efficiencies typically range between 0.2 to 0.7 for a polymeric thin film on a silicon substrate. For a more detailed discussion about the heat transfer between the tip and a substrate, we refer the interested reader to the existing literature ^3,4^.

## A Brief Remark on Reaction Kinetics:

The kinetics of thermally activated mechanisms directly affects the maximum achievable patterning speed in t-SPL and the spatial extent of the reaction as illustrated in Figure 2d in the main text. Due to the broad range of possible thermally driven reactions, we limit the discussion of reaction kinetics to simple first-order reactions and assume a steady state temperature distribution in the sample material around the tip indent. During t-SPL, often a material is thermally degraded, evaporated or chemically converted. The extent of the reaction $\alpha$ increases with time $t$ until all the material has been converted. For a first order reaction, the conversion rate $d\alpha/dt$ can be written as:

$$\frac{d\alpha}{dt}=k\left( 1-\alpha\right), (5)$$

whereby the reaction rate $k$ can often be described by the Arrhenius equation:

$$k=A\cdot\exp\left( -\frac{E_{A}}{k_{B}T} \right).(6)$$

Here, $A$ a pre-exponential factor, $E_{A}$ the activation energy for the reaction, $k_{B}$ the Boltzmann constant, and $T$ is the temperature of the material, which can often be reasonably approximated with an exponentially decaying function $T\left( r \right)=T_{C}\exp\left( -r/\lambda\right)$ ^8,9^. For simplicity, a steady state temperature distribution is assumed, which is justified if the reaction takes considerably longer than the time required to establish a steady-state temperature distribution (see discussion in the previous section). Under these assumptions, the amount of converted material can be expressed as:

$$\alpha=1-\exp\left( -At\cdot exp \left( -\frac{E_{A}}{k_{B}T(r)} \right) \right). (7)$$

In Figure 2e in the main text, a reaction curve obtained from Equation 7 is qualitatively plotted as function of temperature for a given heating time. The gray circles indicate the temperature at which 1% and 99% and the red circle where 50% of the material has been converted. Figures 2f-h in the main manuscript show in general how activation energy, tip-sample contact duration and indentation force affect the temperature at which the thermal conversion takes place. The red curve corresponds to the 50% conversion, and the lower and upper gray curves correspond to the 1% and 99% conversion, respectively. As a general trend, the higher the activation energy the higher is the required temperature to induce a reaction as shown in Figure 2f. Shorter heating times require higher tip temperatures as shown in Figure 2g, which can be a limiting factor in attainable throughput of t-SPL (e.g. for thermally induced crystallization). Another less obvious factor is the indentation force, which can affect the reaction kinetics during t-SPL due to the high pressure which builds up at the tip-sample interface ^10^. A higher force reduces the effective activation energy of the reaction and as a consequence, the same degree of conversion can be obtained with a lower temperature (see Figure 2h).

Note that the model presented here simplifies the complexity of reactions involved during t-SPL. Nevertheless, the discussion allows to qualitatively understand the effect of important experimental parameters on the thermal conversion of a material. A more detailed analysis of the reaction kinetics with heated probes can be found in the literature ^8,10^.

## Some Activation Energies of Thermochemical Reactions used in t-SPL

Table S1 shows some values for thermally triggered reactions of organic materials that are used in t-SPL.

**Table S1 Some activation energies of materials used for t-SPL**

| **Material** | **E_A_ (eV)** | **Ref** |
| --- | --- | --- |
| Cinnamate-Carbamate Polymer | 1.4-1.66 | ^8,11^ |
| Polyphthalaldehyde | 0.98-1.62 | ^6^ |
| MA20 | 1.30 | ^12^ |
| A20 | 1.21 | ^12^ |

References:

1. Dürig, U. Fundamentals of micromechanical thermoelectric sensors. *Journal of Applied Physics* **98**, 044906 (2005).

2. Spieser, M., Rawlings, C., Lörtscher, E., Duerig, U. & Knoll, A. W. Comprehensive modeling of Joule heated cantilever probes. *Journal of Applied Physics* **121**, 174503 (2017).

3. Gotsmann, B., Lantz, M. A., Knoll, A. & Dürig, U. Nanoscale Thermal and Mechanical Interactions Studies using Heatable Probes. in *Nanotechnology* 121–169 (American Cancer Society, 2010). doi:10.1002/9783527628155.nanotech066.

4. Nelson, B. A. & King, W. P. Modeling and Simulation of the Interface Temperature Between a Heated Silicon Tip and a Substrate. *Nanoscale and Microscale Thermophysical Engineering* **12**, 98–115 (2008).

5. Prasher, R. Predicting the Thermal Resistance of Nanosized Constrictions. *Nano Lett.* **5**, 2155–2159 (2005).

6. Holzner, F. Thermal Scanning Probe Lithography using Polyphthalaldehyde. (ETH Zürich, 2013).

7. Yovanovich, M. M., Culham, J. R. & Teertstra, P. Analytical modeling of spreading resistance in flux tubes, half spaces, and compound disks. *IEEE Transactions on Components, Packaging, and Manufacturing Technology: Part A* **21**, 168–176 (1998).

8. Carroll, K. M. *et al.* Fabricating Nanoscale Chemical Gradients with ThermoChemical NanoLithography. *Langmuir* **29**, 8675–8682 (2013).

9. Duvigneau, J., Schönherr, H. & Vancso, G. J. Nanoscale Thermal AFM of Polymers: Transient Heat Flow Effects. *ACS Nano* **4**, 6932–6940 (2010).

10. Raghuraman, S., Elinski, M. B., Batteas, J. D. & Felts, J. R. Driving Surface Chemistry at the Nanometer Scale Using Localized Heat and Stress. *Nano Letters* **17**, 2111–2117 (2017).

11. Wang, D. *et al.* A New AFM-Based Lithography Method: Thermochemical Nanolithography. in *Scanning Probe Microscopy in Nanoscience and Nanotechnology* (ed. Bhushan, B.) 795–811 (Springer Berlin Heidelberg, 2010). doi:10.1007/978-3-642-03535-7_22.

12. Duvigneau, J., Schönherr, H. & Vancso, G. J. Scanning Thermal Lithography of Tailored tert-Butyl Ester Protected Carboxylic Acid Functionalized (Meth)acrylate Polymer Platforms. *ACS Applied Materials & Interfaces* **3**, 3855–3865 (2011).
